# Transcriptomic Analysis of Fermented Chinese Chive Selectively Attenuating Deoxynivalenol-Induced Ovarian Toxicity in Mice

**DOI:** 10.3390/antiox15040442

**Published:** 2026-04-01

**Authors:** Hong Zou, Chun-Yan Qin, Teerath Kumar Suthar, Yupeng Xie, Koroloso Phomane Abednicco, Chun-Feng Wang, Min Kyu Kim, Shu-Min Zhang, Wu-Sheng Sun

**Affiliations:** 1 Jilin Provincial Engineering Research Center of Animal Probiotics, Jilin Provincial Key Laboratory of Animal Microecology and Healthy Breeding, Engineering Research Center of Microecological Vaccines (Drugs) for Major Animal Diseases, Ministry of Education, College of Animal Science and Technology, Jilin Agricultural University, Changchun 130118, China; zouhong@mails.jlau.edu.cn (H.Z.); chunyanqin@mails.jlau.edu.cn (C.-Y.Q.); kumar@mails.jlau.edu.cn (T.K.S.); xieyupeng@mails.jlau.edu.cn (Y.X.); koroloso955@mails.jlau.edu.cn (K.P.A.); wangchunfeng@jlau.edu.cn (C.-F.W.); 2 Sanjiang Laboratory, Changchun 130118, China; 3 University of Chinese Academy of Sciences, Beijing 100049, China; 4 Department of Animal Science and Biotechnology, College of Agriculture and Life Science, Chungnam National University, Daejeon 34134, Republic of Korea; kminkyu@cnu.ac.kr

**Keywords:** deoxynivalenol, fermented Chinese chive, ovarian transcriptomics, oocyte quality, motile cilia, oxidative stress

## Abstract

Deoxynivalenol (DON) is a common mycotoxin linked to ovarian oxidative stress, toxicity, and reduced reproductive performance. Fermented Chinese chive is known for its antioxidant properties and potential reproductive benefits, but their individual and combined effects on ovarian function remain unclear in post-pubertal mice. In this study, a 21-day oral gavage model in female Kunming mice was used to evaluate the effects of DON (2 mg/kg/day), fermented Chinese chive extract (LEEK; 0.2 mL/day), and their combined exposure (LKDON) on ovarian physiology, oocyte quality, and ovarian transcriptomic responses. The results showed that DON exposure significantly reduced the zygote cleavage rate, increased intracellular reactive oxygen species levels, and disrupted oocyte mitochondrial membrane potential. While histological examination revealed disturbed follicular architecture. Transcriptomic hub gene analysis showed that DON exposure down-regulate the key associated with innate immune responses and motile cilia/axonemal structure, including *Rsph4a*, *Drc1*, *Zmynd10*, *Hydin*, and *Tmem212*. In contrast, LEEK alone was associated with immunomodulatory upregulated genes, including *Il5*, *Cd27*, and *Crp*. Interestingly, LKDON and DON comparison revealed upregulation of a motile cilia/axoneme gene network (*Dnah5*, *Dnah11*, *Tekt1*, *Zmynd10*, *Cfap44*, and *Spag6l*), rather than a global reversal of DON-induced changes. Overall, finding suggest that DON disrupts ovarian immune and structural pathways, while fermented Chinese chive provides partial protection by modulating specific biological processes. Further studies are needed to confirm the underlying mechanisms.

## 1. Introduction

Mycotoxin contamination of agricultural commodities represents a major challenge to global food safety and public health [1]. Among these toxins, deoxynivalenol (DON), a trichothecene mycotoxin predominantly produced by *Fusarium graminearum* and *F. culmorum*, is ubiquitously detected in cereal grains such as wheat, maize, and barley [2]. Chronic dietary exposure to DON, even at subclinical levels, is associated with a spectrum of adverse health effects, including anorexia, immunotoxicity, and intestinal barrier dysfunction [3]. Notably, the female reproductive system is highly sensitive to DON. Evidence from rodent and porcine models indicates that DON exposure compromises oocyte quality through multiple interconnected pathways [4]. Mechanistically, DON exerts dual toxicity on oocytes, it directly disrupts meiotic spindle assembly via MAPK inhibition, while simultaneously impairing cytoplasmic maturation through oxidative stress, epigenetic dysregulation, and autophagy-apoptosis imbalance [5,6,7]. Despite this progress, a comprehensive understanding of the precise molecular pathways by which DON compromises oocyte developmental competence, particularly at the level of individual gametes, remains an active area of investigation [8].

Concurrently, there is a paradigm shift towards identifying natural, food-derived compounds capable of mitigating the deleterious effects of environmental toxins. Fermentation, as a bioprocessing technique, can significantly enhance the bioactive profile of plant materials by generating novel metabolites and increasing the bioavailability of polyphenols and antioxidants [9]. Chinese chive (*Allium tuberosum Rottler ex Spreng*) is a traditional herb rich in organosulfur compounds (e.g., allicin and diallyl sulfide) and flavonoids, known for its antimicrobial and antioxidant properties [10]. Fermentation with probiotic strains like *Lactobacillus plantarum* has been shown to enhance the antioxidant capacity and generate beneficial postbiotics in similar plant matrices, suggesting a promising strategy for developing functional ingredients against oxidative damage [11,12].

Traditional toxicological assessments often rely on histological and bulk biochemical analyses of ovarian tissue, which may mask the heterogeneous and cell-type-specific responses of oocytes and their surrounding somatic cells to toxicants [13,14]. The advent of high-throughput omics technologies offers transformative potential. Bulk RNA sequencing of ovarian tissue provides a global view of transcriptional changes across all cell types [15]. However, this approach cannot resolve cell-type-specific responses, as molecular changes in whole ovarian tissue reflect a mixture of oocytes, granulosa cells, and other somatic cells. Thus, while the transcriptomic analysis identifies key pathways and hub genes, their precise cellular origin requires validation with higher-resolution techniques. Integrating these approaches with classical physiological and cellular assays provides a robust strategy for mechanistic discovery.

Therefore, this study aimed to investigate the effects of deoxynivalenol (DON) on ovarian function and to determine whether fermented Chinese chive extract can modulate DON-associated molecular alterations in post-pubertal mice. Specifically, we characterized ovarian transcriptomic responses to DON, identified key genes and pathways involved in ovarian dysfunction, and evaluated the impact of fermented Chinese chive under co-exposure conditions. Our findings indicate that DON-induced reproductive toxicity is associated with suppressed immune responses and reduced expression of ciliary motility-related genes, whereas fermented Chinese chive partially mitigates these effects and improves reproductive performance.

## 2. Materials and Methods

### 2.1. Ethics Statement

All animal experiments were performed in strict compliance with the guidelines of the Institutional Animal Care and Use Committee (IACUC) of Jilin Agricultural University. The experimental protocol was formally reviewed and approved by the IACUC (Approval No. 20240428003).

### 2.2. Fermented Chinese Chive Extract and DON Preparation

Fermented Chinese chive extract and yeast powder were provided by the Institute of Biological Resources, Jiangxi Academy of Sciences. Fresh Chinese chives (*Allium tuberosum*) were juiced and filtered to obtain the filtrate, which was diluted with distilled water at a ratio of 1:4 [16]. The diluted juice was inoculated with *Lactobacillus plantarum* (1 × 10^6^ CFU/mL) and fermented at 37 °C for 24 h. After fermentation, the extract was stored at 4 °C until use. The analytical standard of Deoxynivalenol (DON) was purchased from GlpBio (Montclair, CA, USA). DON was dissolved in dimethyl sulfoxide (DMSO) and diluted with physiological saline to obtain the required concentrations. The final DMSO concentration did not exceed 0.1% [17,18].

### 2.3. Untargeted Metabolomic Profiling by UPLC-ESI-MS/MS

Fermented Chinese chive extract was analyzed using an ultra-performance liquid chromatography–electrospray ionization tandem mass spectrometry (UPLC–ESI–MS/MS) system consisting of an ExionLC™ AD UPLC system (SCIEX, Framingham, MA, USA) coupled with a QTRAP mass spectrometer (SCIEX, Framingham, MA, USA). Chromatographic separation was performed using an Agilent SB-C18 column (1.8 μm, 2.1 × 100 mm). The mobile phase consisted of solvent A (water containing 0.1% formic acid) and solvent B (acetonitrile containing 0.1% formic acid). The gradient elution program was as follows: initial conditions of 95% A and 5% B; a linear gradient to 5% A and 95% B over 9 min; maintained at 5% A and 95% B for 1 min; returned to 95% A and 5% B within 1.1 min; and equilibrated for 2.9 min. The flow rate was set at 0.35 mL min^−1^, the column temperature was maintained at 40 °C, and the injection volume was 2 μL. The eluent was directly introduced into an electrospray ionization (ESI) source coupled to a triple quadrupole–linear ion trap mass spectrometer (QTRAP-MS) operating in tandem mass spectrometry mode.

### 2.4. Animals, Experimental Design, Mating Procedure, and Sample Collection

Female Kunming mice (closed colony, 6–7 weeks old, 30–40 g) were obtained from Beijing Vital River Laboratory Animal Technology Co., Ltd., Beijing, China. Mice were housed under specific pathogen-free (SPF) conditions at 22–25 °C with 40–60% relative humidity and a 12 h light/dark cycle, with free access to standard chow and water. After a one-week acclimation period, 80 mice were randomly assigned to four groups (*n* = 20 per group): control (CTRL), deoxynivalenol (DON; 2 mg/kg body weight/day), fermented Chinese chive extract (LEEK; 0.2 mL/day), and combined treatment (LKDON). DON and LEEK were administered by oral gavage for 21 consecutive days. To ensure consistent handling and liquid volume among groups, each mouse received two daily gavages separated by at least 4 h. The CTRL group received normal saline (0.2 mL per gavage), the DON group received DON solution (0.2 mL) followed by saline (0.2 mL), the LEEK group received LEEK extract (0.2 mL) followed by saline (0.2 mL), and the LKDON group received DON solution (0.2 mL) followed by LEEK extract (0.2 mL), resulting in a total daily volume of 0.4 mL per mouse. Body weight was recorded at the start of acclimation, immediately before the first gavage (Day 0), and every three days thereafter. Final body weight was measured 24 h after the last gavage prior to euthanasia, and body weight gain was calculated as the difference between final weight and Day 0 weight. Ovarian weights were recorded as both absolute weight (mg) and relative weight (mg/g body weight). Following treatment, mice underwent superovulation. Females received an intraperitoneal injection of pregnant mare serum gonadotropin (PMSG, 5 IU), followed 46–48 h later by human chorionic gonadotropin (hCG, 5 IU). For mating experiments, females were paired with proven males (1:1) immediately after hCG injection. Successful mating was confirmed by the presence of a vaginal plug the following morning. Plug-positive females were euthanized by cervical dislocation 18–22 h after hCG administration for collection of zygotes from the oviduct ampulla. Ovaries, oocytes, and zygotes were collected under sterile conditions. Oviducts were placed in pre-warmed PBS, and cumulus–oocyte complexes (COCs) or zygotes were released from the ampulla using fine forceps under a stereomicroscope. Cumulus cells were removed using M2 medium containing hyaluronidase (500 U/mL), followed by washing in fresh M2 medium. Oocytes and zygotes were counted and evaluated using an inverted microscope (Leica DMI 1, Wetzlar, Germany) and maintained in pre-warmed M2 medium at 37 °C until further analysis. For transcriptomic analysis, whole ovaries were dissected free of surrounding tissues, briefly rinsed in ice-cold PBS, snap-frozen in liquid nitrogen, and stored at −196 °C until RNA extraction.

### 2.5. Cleavage Rate Counting Method

After hyaluronidase treatment, zygotes were immediately transferred through three 50 μL drops of fresh, pre-warmed M2 medium in a 35 mm Petri dish. In each drop, zygotes were gently pipetted up and down 2–3 times using a fine glass pipette to remove residual enzyme and cellular debris before being moved to the next drop. Following the final wash, cleaned zygotes were transferred into pre-equilibrated M16 culture drops overlaid with mineral oil (equilibrated at 37 °C in 5% CO_2_ for ≥4 h) and cultured for 12 h. After incubation, zygotes were examined microscopically, and the number reaching the two-cell stage was recorded. For each independent experiment, the cleavage rate was calculated as: (number of two-cell zygotes/total number of cultured fertilized eggs) × 100%. The cleavage rate was calculated from at least three independent experiments and is presented as mean ± SD.

### 2.6. Histological Analysis

For morphological analysis, ovarian tissues were fixed in 4% paraformaldehyde, embedded in paraffin, and serially sectioned at a thickness of 5 μm. The sections were then stained with hematoxylin and eosin (H&E) following standard protocols. Follicle classification was performed based on the morphological criteria established in previous studies [19,20]. Specifically, follicles were categorized as follows: Primordial follicles: Characterized by an oocyte surrounded by a single layer of flattened (squamous) pre-granulosa cells. Primary follicles: Identified by an oocyte surrounded by a single layer of cuboidal granulosa cells. Secondary follicles: Defined by an oocyte surrounded by two or more layers of cuboidal granulosa cells, with no visible antrum formation. Mature follicles: Recognized by the presence of a defined antrum (fluid-filled cavity) within the granulosa cell layers.

### 2.7. ROS and Mitochondrial Membrane Potential Assays

Intracellular reactive oxygen species (ROS) levels in mouse oocytes were assessed using a fluorescent ROS detection probe (Beyotime C1035 Mito-Tracker Red CMXRos, Beyotime Biotechnology Co., Ltd., Shanghai, China) according to the manufacturer’s instructions. Mitochondrial membrane potential was evaluated using the Beyotime C2006 JC-1 mitochondrial membrane potential assay kit (Beyotime Biotechnology Co., Ltd., Shanghai, China), and fluorescence intensity was analyzed using a Leica DMI8 inverted fluorescence microscope (Leica Microsystems GmbH, Wetzlar, Germany).

### 2.8. RNA Extraction and Library Preparation of Ovarian Tissues

Ovarian tissues from four randomly selected biological replicates per group were used for transcriptomic analysis. Total RNA was isolated using Trizol Reagent (Invitrogen Life Technologies, Carlsbad, CA, USA). RNA concentration and purity were assessed using a NanoDrop spectrophotometer (Thermo Scientific, Waltham, MA, USA), and RNA integrity was evaluated using an Agilent 2100 Bioanalyzer (Agilent Technologies, Santa Clara, CA, USA). All samples used for library construction had RNA integrity number (RIN) values > 8.0. Approximately 3 µg of total RNA from each sample was used for library preparation. mRNA was purified from total RNA using poly-T oligo-attached magnetic beads and then fragmented using divalent cations under elevated temperature in Illumina proprietary fragmentation buffer. First-strand cDNA was synthesized with random oligonucleotides and SuperScript II reverse transcriptase, followed by second-strand cDNA synthesis using DNA Polymerase I and RNase H. The remaining overhangs were converted into blunt ends via exonuclease/polymerase activity. After adenylation of the 3′ ends of the DNA fragments, Illumina paired end (PE) adapter oligonucleotides were ligated for hybridization. cDNA fragments of 400–500 bp in length were selected and purified using the AMPure XP system (Beckman Coulter, Beverly, CA, USA). DNA fragments with ligated adapters were selectively enriched using Illumina PCR Primer Cocktail in a 15-cycle PCR reaction. The PCR products were purified using the AMPure XP system. Library quality control was performed as follows: library concentration was quantified using the Qubit fluorometer (Thermo Fisher Scientific, Waltham, MA, USA) and real-time PCR (qPCR), and library fragment size distribution was assessed using the Agilent High Sensitivity DNA assay on a Bioanalyzer 2100 system (Agilent Technologies).

### 2.9. Bioinformatics Analysis

The mRNA expression of eight sequencing libraries were analyzed using RNA-seq. Raw FASTQ reads were subjected to quality control and filtering using fastp (v0.22.0) to remove low-quality reads and adaptor sequences [21]. The clean reads were then aligned to the *Mus musculus* reference genome (GRCm39, GCA_000001635.9) using HISAT2 (v2.1.0) [22]. Gene expression levels were quantified using HTSeq (v0.9.1) and read counts were normalized as fragments per kilobase of transcript per million mapped reads (FPKM) for visualization purposes. Differentially expressed genes (DEGs) were identified using DESeq2 (v1.38.3) with thresholds of |log_2_ fold change| > 1 and a Benjamini–Hochberg adjusted *p*-value (FDR) < 0.05 were considered significantly differentially expressed [23]. Venn diagrams were generated using the R (v4.5.2) statistical environment [24]. Bidirectional hierarchical clustering of DEGs was performed using the pheatmap package (v1.0.12) in R [25]. Functional annotation was conducted using BioMart (v2.66.2) for gene identifier conversion [26]. Gene Ontology (GO) enrichment analysis was performed using the GO package (v2.50.0) [27], and Kyoto Encyclopedia of Genes and Genomes (KEGG) pathway enrichment analysis was conducted using clusterProfiler (v4.6.0) [28]. Protein–protein interaction (PPI) networks were constructed using the STRING database [29]. To reduce network complexity, DEGs with |log_2_ fold change| > 2 and adjusted *p*-value < 0.05 were selected for protein–protein interaction analysis and visualization in Cytoscape (v3.10.2) [30].

### 2.10. Molecular Docking

The two-dimensional structures of small-molecule ligands were obtained from the PubChem database and converted into three-dimensional forms using ChemOffice (v22.0.0), then saved in mol2 format. Crystal structures of target proteins were retrieved from the RCSB Protein Data Bank. Using PyMOL (v3.1), water molecules and modifications were removed, and the proteins were saved in PDB format. Protein and ligand structures were prepared using AutoDock Tools (v1.5.7) by adding hydrogen atoms, removing water molecules, and defining ligand flexibility. A docking grid was set with appropriate coordinates and size. Molecular docking was performed using AutoDock Vina (v1.1.2) to predict binding modes and interaction energies, and the best pose was selected based on the lowest binding affinity. Docked complexes were visualized and analyzed using PyMOL (v2.3) and Discovery Studio 2019 Client (v19.1.0).

### 2.11. Validation of DEGs

To validate the RNA-seq results, six genes (*CCL11*, *ZMYND10*, *PTGS2*, *RSPH4A*, *PLA2G4B*, and *CD27*) were selected for RT–qPCR analysis. Four biological replicates per group were analyzed, with three technical replicates per sample. Total RNA was obtained from the same samples used for RNA-seq. Primers were designed using NCBI Primer-BLAST (https://www.ncbi.nlm.nih.gov/tools/primer-blast/index.cgi?GROUP_TARGET=on, accessed on 30 January 2026) and Primer 5.0. RT–qPCR was performed under previously optimized conditions, with a final primer concentration of 10 μmol/μL. *GAPDH* was used as the reference gene. Gene expression was analyzed using GraphPad Prism 10, and results are presented as mean ± standard error (SE). Primer sequences are listed in (Table 1).

### 2.12. Statistics Analysis

All data are presented as mean ± standard deviation (SD), with units indicated in the corresponding figure legends and table footnotes. Statistical analyses were conducted using GraphPad Prism 10. Data normality and homogeneity of variances were assessed using the Shapiro–Wilk and Bartlett’s tests, respectively. As these assumptions were satisfied, differences among groups were evaluated by one-way analysis of variance (ANOVA) followed by Tukey’s honestly significant difference (HSD) post hoc test. For embryo/oocyte data, the experimental unit was the mouse, with values averaged per animal (*n* = number of animals per group). Proportional data (cleavage rates) were analyzed using a generalized linear model with a binomial distribution and logit link function. A *p*-value < 0.05 was considered statistically significant.

## 3. Results

### 3.1. Effects of DON, LEEK, LKDON on Body Weight Gain and Ovarian Weight in Mice

**Figure 1:**
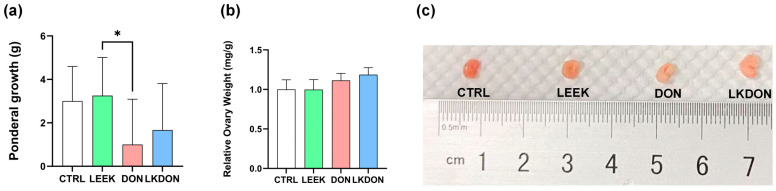
Effects of fermented Chinese chive extract on body weight gain and relative ovarian weight in DON-exposed mice. (CTRL, control group; LEEK, fermented Chinese chive extract group; DON, deoxynivalenol group; LKDON, combined treatment group). (**a**) Body weight gain over the 21-day gavage period. (**b**) Relative ovarian weight. (**c**) Representative images of ovarian size from each group (from left to right: CTRL, LEEK, DON, and LKDON, respectively). * *p* < 0.05.

Body weight gain during the 21-day gavage period is shown in Figure 1a. No significant differences in feed intake, diarrhoea, or other adverse health effects were observed among the experimental groups during the treatment period. Mice in the DON-treated group exhibited lower body weight gain compared with the other groups. Supplementation with fermented Chinese chive extract increased body weight gain relative to the DON group; however, this increase was not statistically significant compared with the control group. A significant difference in body weight gain was observed between the LEEK and DON (*p* ≤ 0.05). In contrast, relative ovarian weight did not differ significantly among the experimental groups (Figure 1b).

### 3.2. Histological Analysis of Mouse Ovaries by H&E Staining

**Figure 2:**
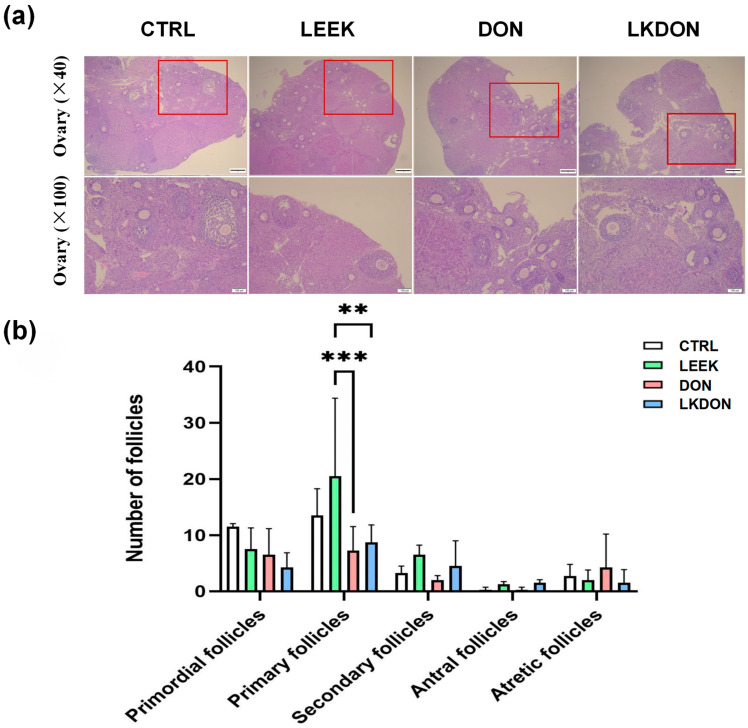
Histological analysis of mouse ovarian tissue by H&E staining. (CTRL, control group; LEEK, fermented Chinese chive extract group; DON, deoxynivalenol group; LKDON, combined treatment group). (**a**) Representative H&E-stained images showing ovarian morphology in each group. Upper panels, ×40 magnification; lower panels, ×100 magnification of the boxed areas in the upper panels. Scale bars: 50 µm (upper), 100 µm (lower). (**b**) Quantification of follicle counts at different developmental stages in each group. ** *p* < 0.01, *** *p* < 0.001.

In the CTRL and LEEK groups, ovarian follicles exhibited intact morphology, with well-organized granulosa cell layers and follicles at different developmental stages. In contrast, ovaries from the DON-treated group displayed irregular follicular morphology, loosely arranged granulosa cells, and a reduced gap between the granulosa cell layer and the follicular antrum. Quantitative analysis revealed no significant differences in the numbers of primordial, secondary, or mature follicles among the groups. However, the number of primary follicles in the LEEK group was significantly higher than that in the DON and LKDON groups (*p* ≤ 0.01) (Figure 2). These results suggest that fermented Chinese chive extract promotes the development of primary follicles and partially alleviates the toxic effects of DON under co-treatment conditions.

### 3.3. Effects of DON, LEEK, LKDON on Cleavage Rate (Early Embryonic Development) in Mice

As shown in Table 2, the mean zygote cleavage rate in the DON group (27.08%) was significantly lower than that in the CTRL group (56.89%, *p* < 0.001). Co-treatment with fermented Chinese chive extracts partially rescued this defect, with the LKDON group exhibiting a significantly higher mean cleavage rate (45.20%) compared with the DON group (*p* < 0.001), although it remained lower than that of the CTRL group (*p* < 0.05). No significant difference was observed between the CTRL and LEEK groups (51.18%, *p* > 0.05), nor between the LEEK and LKDON groups (*p* > 0.05). These results indicate that DON exposure significantly impairs oocyte developmental competence, as reflected by reduced zygote cleavage rates, and that co-treatment with fermented Chinese chive extract partially alleviates this DON-induced defect.

### 3.4. ROS and JC-1 Mitochondrial Staining of Mouse Oocyte

**Figure 3:**
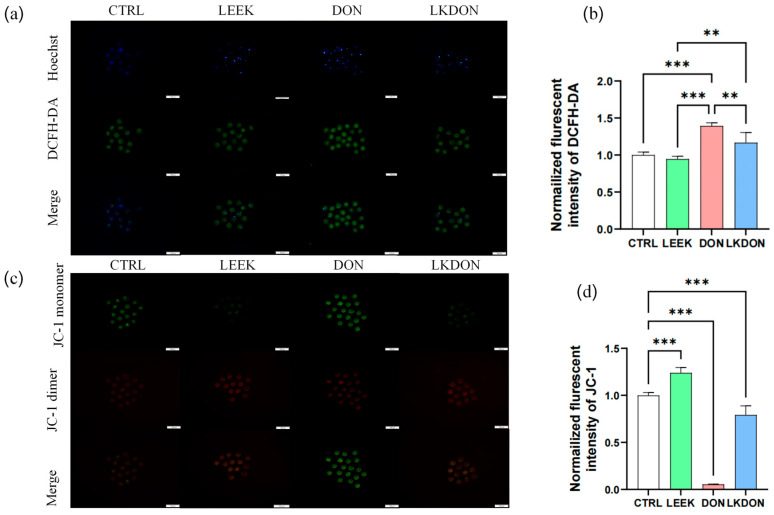
Fermented Chinese chive extract alleviates DON-induced oxidative stress and mitochondrial dysfunction. (CTRL, control group; LEEK, fermented Chinese chive extract group; DON, deoxynivalenol group; LKDON, combined treatment group). (**a**) Representative fluorescence images of DCFH-DA staining for intracellular ROS levels in each group. A scale bar of 100 µm is included for reference. (**b**) Quantification of normalized DCFH-DA fluorescence intensity. Data are presented as mean ± SEM. (**c**) Representative fluorescence images of JC-1 staining showing mitochondrial membrane potential in each group. A scale bar of 100 µm is included for reference. (**d**) Quantification of normalized JC-1 mitochondrial membrane potential fluorescence intensity. ** *p* < 0.01; *** *p* < 0.001.

As shown in Figure 3, exposure to DON significantly increased the normalized fluorescent intensity of DCFH-DA, an indicator of intracellular ROS levels, compared with the CTRL, LEEK, and LKDON groups (*p* ≤ 0.01), indicating elevated intracellular ROS production. To assess whether these changes were associated with altered mitochondrial function, mitochondrial membrane potential (ΔΨm) was assessed using JC-1 staining. In the DON-treated group, a significant decrease in the red/green fluorescence ratio was observed (*p* ≤ 0.01), reflecting reduced ΔΨm, which was partially restored in the LKDON group. Taken together, these data suggest that DON exposure disrupts cellular redox balance, as reflected by increased ROS levels and reduced mitochondrial membrane potential in mouse oocytes under the experimental conditions used. The protective effect of fermented Chinese chive extract against DON-induced mitochondrial depolarization indicates its potential to alleviate DON-associated cellular dysfunction (Figure 3).

### 3.5. Transcriptomics Analysis of Mouse Ovaries

#### 3.5.1. Transcriptome Sequencing and Read Distribution Summary

RNA-seq generated ~42–54 million reads per library across all groups (n = 4 per group) with high mapping rates (88.7–94.0%) and 82.6–89.4% uniquely mapped reads. Most reads aligned to exonic regions (85.2–93.8%), with low intronic and intergenic proportions, indicating high library integrity and effective enrichment of mature transcripts. Proper pairing, balanced strand distribution, and consistent splicing rates across samples confirmed reliable transcript detection and data quality suitable for downstream analyses (Table S1).

#### 3.5.2. Comparative Transcriptomics Analysis of Mouse Ovaries

**Figure 4:**
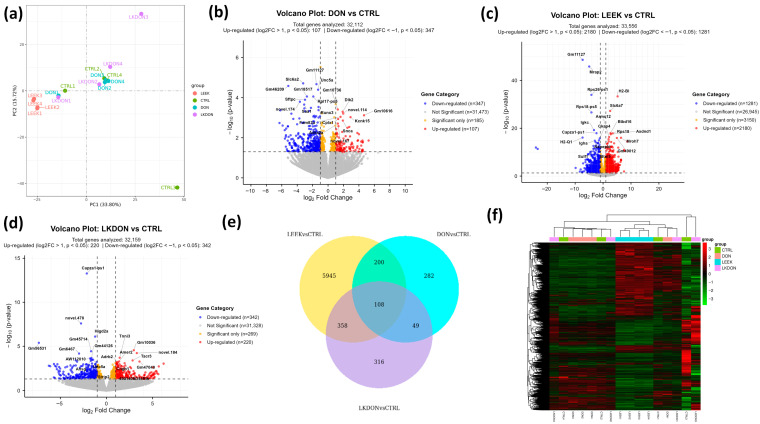
Differential gene expression analysis of mouse ovaries treated with deoxynivalenol (DON), fermented Chinese chive (LEEK), and a mixture of fermented Chinese chive and deoxynivalenol (LKDON) compared to the control (CTRL) group. (**a**) PCA plot showing distinct clustering of the treatment groups, with PC1 explaining 33.80% and PC2 explaining 15.27% of the variance. (**b**) Volcano plot of DON vs. CTRL showing up-regulated genes (red), down-regulated genes (blue), and significant genes with small fold changes (yellow) from 32,112 genes. (**c**) Volcano plot of LEEK vs. CTRL with 33,556 genes analyzed, highlighting up-regulated (red), down-regulated (blue), and significant genes with small fold changes (yellow). (**d**) Volcano plot of LKDON vs. CTRL, analyzing 32,159 genes, with up-regulated genes in red, down-regulated genes in blue, and significant genes with small fold changes in yellow. (**e**) Venn diagram of multiple comparisons. (**f**) Cluster heatmap trend of expressed genes in different samples of each group.

We conducted a differential gene expression (DEGs) analysis to examine the molecular effects of deoxynivalenol (DON), fermented Chinese chive (LEEK), and their combined treatment (LKDON) relative to the control (CTRL) group in the ovarian tissues of mice (Table S2). The Principal Component Analysis (PCA) plot revealed clear separation among the treatment groups, with the first principal component (PC1) accounting for 33.80% of the variance and the second principal component (PC2) showing 15.27%. This indicates distinct gene expression profiles across experimental groups (Figure 4a). In the DON vs. CTRL comparison, 32,112 genes were analyzed, among them, 107 genes were significantly up-regulated (log2 fold change > 1, *p* < 0.05) and 347 genes were significantly down-regulated (log2 fold change < −1, *p* < 0.05) (Figure 4b). In the LEEK vs. CTRL comparison, a total of 33,556 genes were analyzed, with 2180 genes significantly up-regulated and 1281 significantly down-regulated (Figure 4c). In the LKDON vs. CTRL comparison, 220 genes were significantly up-regulated, and 342 genes were significantly down-regulated (Figure 4d). Venn analysis showed that most DEGs of LEEK vs. CTRL contained the highest number of unique DEGs, followed by LKDON and DON. A core set of 108 genes was shared across all comparisons, indicating common transcriptional responses to treatment (Figure 4e). Clustered heatmap of expressed genes showed strong within-group similarity and distinct expression patterns between treatment and control groups. LEEK and LKDON exhibited partially similar gene expression profiles, while DON displayed a more distinct transcriptional signature (Figure 4f). Overall, results demonstrate that each treatment, DON, LEEK, and LKDON, induces significant changes in gene expression compared to the control, with varying degrees of up-regulation and down-regulation.

#### 3.5.3. Functional Pathway and Gene Ontology Analysis of Differential Gene Expression in Mouse Ovaries Treated with DON, LEEK, and LKDON

**Figure 5:**
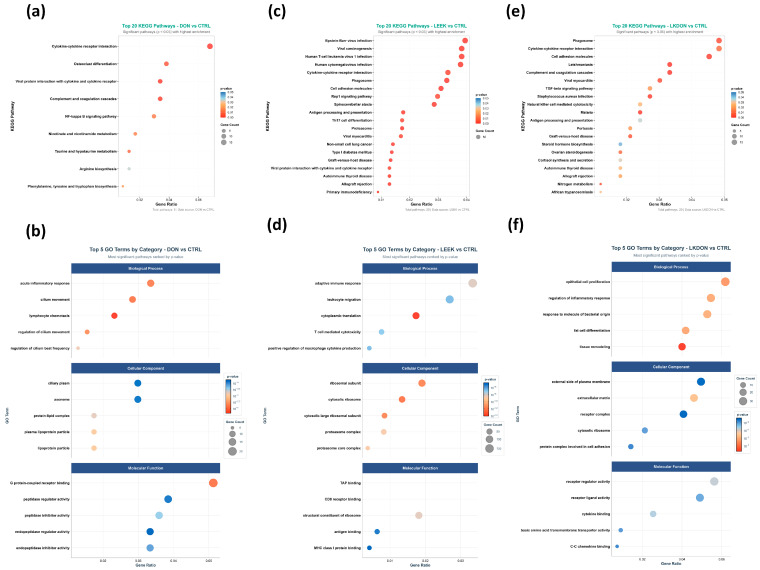
Functional enrichment of ovarian DEGs in female mice after 21-day gavage with deoxynivalenol (DON), fermented Chinese chive extract (LEEK), or co-exposure (LKDON), compared with controls (CTRL). (**a**,**b**) Top KEGG pathways (top 20) and GO terms (top 5 per category) for DON vs. CTRL. (**c**,**d**) Corresponding KEGG and GO enrichment for LEEK vs. CTRL. (**e**,**f**) Corresponding KEGG and GO enrichment for LKDON vs. CTRL. Enrichments are shown by gene count and enrichment ratio.

Kyoto Encyclopedia of Genes and Genomes (KEGG) pathways and Gene Ontology (GO) term enrichment analyses of differentially expressed genes were performed to investigate the biological impacts of deoxynivalenol (DON), fermented Chinese chive (LEEK), and a combination of both (LKDON) treatments compared to the control (CTRL) group in mouse ovaries (Figure 5). KEGG pathway analysis revealed that genes altered by DON exposure were enriched in immune-related pathways, including cytokine-cytokine receptor interactions (*Il20ra*, *Eda2r*, *Cxcl16*, *Il10ra*, *Tnfsf18*, *Acvr1c*, *Cxcl14*, *Ifnlr1*, *Tnfsf14*, *Ackr3*, *Il4ra*, *Ccl8*, *Tnfsf13b*, *Il1rl1*, *Ccl11*, *Csf1r*), complement and coagulation cascades (*C4b*, *A2m*, *Serpinc1*, *F3*, *C5ar1*, *Serpina5*, *C1qa*, *C7*), and osteoclast differentiation (*Socs1*, *Gm12791*, *Fcgr3*, *Pira2*, *Sirpb1b*, *Ncf2*, *Csf1r*, *Map3k14*, *Sirpb1a*), as well as metabolic pathways such as taurine and hypotaurine metabolism (*Ggt1*, *Fmo4*, *Baat*) and the NF-κB signaling pathway (*Eda2r*, *Tnfsf14*, *Tnfsf13b*, *Map3k14*, *Edaradd*, *Prkcb*, *Lat*) (Figure 5a; Table S3). GO term analysis showed enrichment in biological processes including lymphocyte chemotaxis, cilium movement, and acute inflammatory response. Altered cellular components included the ciliary plasma membrane, and enriched molecular functions included peptide receptor activity (Figure 5b). For the LEEK group, KEGG analysis revealed enrichment in pathways such as Th17 cell differentiation, cytokine-cytokine receptor interactions, the Rap1 signaling pathway, and natural killer cell-mediated cytotoxicity (Figure 5c; Table S4). GO term analysis showed enrichment in biological processes including cytoplasmic translation, adaptive immune response, and T cell-mediated cytotoxicity. Enriched cellular components included the ribosomal subunit, and enriched molecular functions included TAP binding (Figure 5d). In the LKDON group, KEGG analysis showed enrichment in cytokine-cytokine receptor interactions, primary immunodeficiency, and Fc gamma R-mediated phagocytosis (Figure 5e; Table S5). GO term analysis indicated enrichment in epithelial cell proliferation, regulation of inflammatory response, and response to bacterial origin. Enriched cellular components included the external side of the plasma membrane, and enriched molecular functions included receptor ligand activity (Figure 5f).

#### 3.5.4. Identification of Hub Genes from DEGs

**Figure 6:**
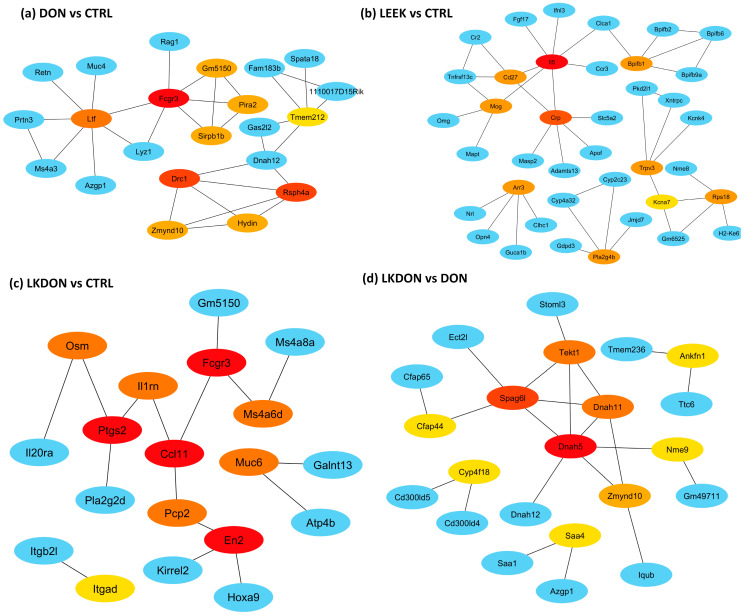
Hub-gene protein–protein interaction (PPI) networks across treatment comparisons in mouse ovary. PPI networks were constructed from differentially expressed genes (DEGs) for each comparison and visualized to those showing high degree of centrality between hub genes and their first genes within network genes. Among illustration panel (**a**) DON vs. CTRL, (**b**) LEEK vs. CTRL, (**c**) LKDON vs. CTRL, and (**d**) LKDON vs. DON showing the hub genes of DEGs of these comparisons. Node color denotes degree of connectivity with red dark, yellow to light yellow indicates higher to lower degree of connectivity among hub nodes, with red representing the most highly connected hubs in each network. Blue nodes indicate first-neighbor genes (direct interaction partners) connected to the hubs. Edges represent known or predicted protein–protein associations within the network.

To identify key regulators underlying treatment-associated ovarian transcriptomic changes, we constructed protein–protein interaction (PPI) networks from differentially expressed genes (DEGs) and defined hub genes by node centrality (Figure 6). In the DON vs. CTRL comparison, hub genes were uniformly downregulated and enriched for innate immune/myeloid markers (*Fcgr3*, *Ltf*, *Sirpb1b*, *Pira2*) as well as genes typically associated with motile cilia/axoneme structure (*Rsph4a*, *Drc1*, *Zmynd10*, *Hydin*, *Tmem212*), indicating coordinated suppression of immune-associated transcripts in response to DON exposure (Figure 6a). In the LEEK vs. CTRL comparison, hub genes were uniformly upregulated, including immune/response-associated genes (*Il5*, *Cd27*, *Crp*) and signaling/lipid mediator-related genes (*Pla2g4b*), consistent with an immunomodulatory transcriptional response to fermented Chinese chive alone (Figure 6b). In the LKDON vs. CTRL comparison, hub genes exhibited mixed directional changes: (*En2*, *Muc6*, *Itgad*) were upregulated, whereas inflammatory and chemokine/prostaglandin-associated mediators (*Ptgs2*, *Ccl11*, *Osm*, *Il1rn*) and the innate immune marker Fcgr3 were downregulated, suggesting selective attenuation of inflammatory signaling during co-exposure while elements of the DON-associated immune signature persisted (Figure 6c). Between-treatment comparisons further refined these patterns. In the DON vs. LEEK comparison, hub genes were dominated by a cytotoxic lymphocyte module (*Ccl5*, *Cd8a*, *Cxcr6*, *Gzma*, *Cd3d*, *Cd3g*, *Cd5*), indicating a stronger T cell/NK-like immune signature under DON relative to LEEK, whereas *Cd27* was comparatively higher in LEEK. In the LKDON vs. DON comparison, hub genes were predominantly upregulated in the LKDON group and mapped to a motile cilia/axoneme-associated network (*Dnah5*, *Dnah11*, *Tekt1*, *Zmynd10*, *Cfap44*, *Spag6l*, *Nme9*), while *Cyp4f18* was downregulated (Figure 6d).

#### 3.5.5. DEG RT-qPCR

**Figure 7:**
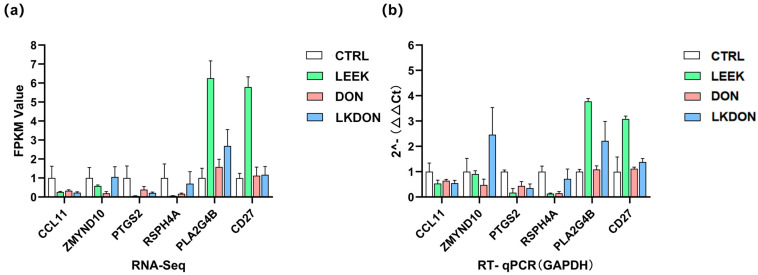
Validation of gene expression is related to early ovarian development. (**a**) Transcript abundance measured by RNA-seq for selected target genes. (**b**) Relative mRNA expression levels determined by RT-qPCR, normalized to GAPDH.

We randomly selected the six genes (*CCL11*, *ZMYND10*, *PTGS2*, *RSPH4A*, *PLA2G4B*, and *CD27*) for RT-qPCR validation. The expression trends observed by qPCR were highly consistent with the transcriptome sequencing data, strongly confirming the high accuracy, reliability, and reproducibility of the transcriptome sequencing results (Figure 7a,b).

### 3.6. Chemical Profiling of Fermented Chinese Chive Extract and Molecular Docking with Target Proteins

**Figure 8:**
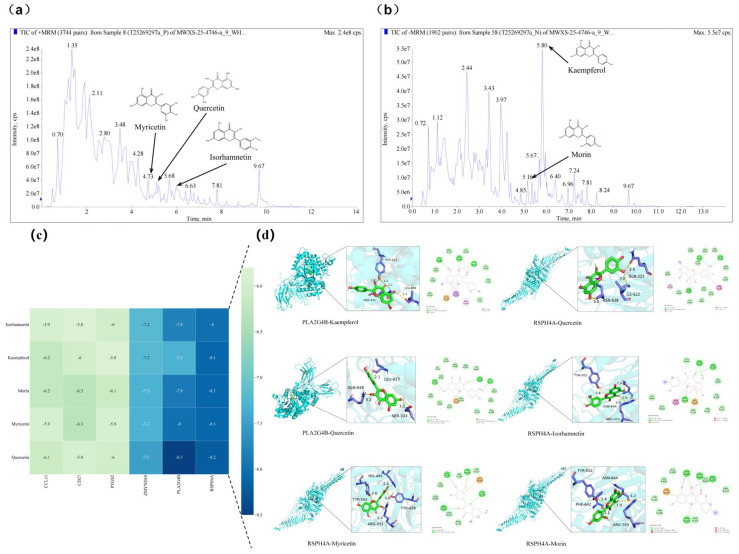
Total ion current (TIC) chromatograms of fermented Chinese chive extract obtained by UHPLC-QTOF-MS in (**a**) positive and (**b**) negative electrospray ionization (ESI) modes. Five flavonoids—myricetin, morin, quercetin, kaempferol, and isorhamnetin—were identified by matching their retention times and mass spectral characteristics with those of authentic reference standards. Their chemical structures and trivial names are labeled on the corresponding chromatographic peaks for clarity. Molecular docking analysis of bioactive flavonols from fermented Chinese chive with key target proteins. (**c**) Heatmap of binding energies (kcal/mol) for interactions between five major flavonols (isorhamnetin, kaempferol, morin, myricetin, and quercetin) and six key target proteins (*CCL11*, *CD27*, *PTGS2*, *ZMYND10*, *PLA2G4B*, and *RSPH14A*). The color gradient from light to dark blue represents decreasing binding energy, indicating progressively stronger binding affinity. (**d**) Representative binding conformations of flavonols docked with PLA2G4B and RSPH14A. For each complex, the left panel shows a 3D ribbon diagram with a magnified view of the binding pocket, and the right panel presents a 2D interaction map. Key hydrogen bonds and hydrophobic interactions are indicated, with bond distances labeled in Ångströms (Å).

Total ion current (TIC) chromatograms of fermented Chinese chive extract revealed the presence of several flavonoid compounds detected under both positive and negative electrospray ionization (ESI) modes (Figure 8). In the positive ionization mode, myricetin, quercetin, and isorhamnetin were observed at retention times of 3.68 min, 5.16 min, and 6.63 min, respectively (Figure 8a). In the negative ionization mode, kaempferol and morin were detected at retention times of 5.80 min and 5.67 min, respectively (Figure 8b). The chromatographic peaks corresponding to these compounds are labelled with their respective chemical structures and compound names in Table S6.

Based on these identified flavonols, molecular docking of the identified flavonols with *PLA2G4B* and *RSPH4A* were performed to evaluate their binding affinities. In molecular docking, a lower binding free energy signifies a stronger ligand-target interaction, indicating enhanced affinity and complex stability. All evaluated compounds exhibited binding energies below −5.0 kcal/mol with both *PLA2G4B* and *RSPH4A*. Notably, quercetin displayed the highest affinity for *PLA2G4B* (−8.5 kcal/mol) and the most favorable interaction with *RSPH4A* (−8.2 kcal/mol) (Figure 8c). Analysis of the binding poses revealed that these interactions are stabilized by hydrogen bonding and hydrophobic contacts between the ligands and key residues within the active pockets (Figure 8d). These results suggest that the tested compounds can stably interact with and potentially modulate the function of the target proteins, providing direct structural evidence for the mechanism by which LEEK alleviates DON-induced reproductive toxicity.

## 4. Discussion

Deoxynivalenol exposure impaired oocyte developmental competence, increased oxidative stress, disrupted mitochondrial function, and altered ovarian morphology. Transcriptomic analysis further revealed coordinated alterations in immune-related and epithelial structural pathways. Fermented Chinese chive extracts alone induced an immunomodulatory transcriptional profile, whereas co-exposure selectively modulated DON-associated gene expression rather than producing a global reversal of DON-induced transcriptional changes.

DON is a ribosome-targeting trichothecene that triggers the ribotoxic stress response and activates MAPK signaling, which then reshapes stress/inflammation transcription programs [31,32]. DON has also been reported to induce ovarian toxicity and uterine damage in mice [33]. Its exposure is widely reported to induce oxidative stress and can disturb mitochondrial dynamics/signaling [34]. In vivo, DON-exposed mouse ovaries show follicular atresia, nuclear pyknosis, reduced *PCNA/Ki67*, impaired oocyte number and maturation, and disrupted expression of steroid hormone-related genes, indicating broad ovarian dysfunction [35]. Our findings further suggest that DON disrupts redox balance, mitochondrial function, and alters ovarian function.

Typically, DON activates MAPK and NF-κB pathways and induces pro-inflammatory gene expression in classical myeloid target cells such as macrophages and monocytes, often producing a strong innate immune signature [36]. In contrast, our study showed the uniform downregulation of myeloid/innate markers (*Fcgr3*, *Ltf*, *Sirpb1b*, and *Pira2*) in ovarian tissue, suggesting that chronic or tissue-specific DON exposure may instead suppress local immune-associated transcription. This pattern may reflect depletion or functional silencing of ovarian myeloid-like cells or their signaling pathways [37]. Looking at the gene level, *Fcgr3* encodes a low-affinity Fcγ receptor (CD16) that binds IgG and mediates functions such as phagocytosis and antibody-dependent cellular cytotoxicity in macrophages, neutrophils and NK cells [38]. Ovarian expression showed the activation state of myeloid/NK-like cells in the ovarian microenvironment and downregulation suggests reduced local immune effector activity following DON exposure [37]. *Ltf* encodes lactoferrin, an iron-binding glycoprotein with antimicrobial and immunomodulatory properties, expressed in neutrophils and mucosal epithelia and detectable in follicular fluid [39]. In the ovary, lactoferrin has been linked to modulation of granulosa-cell cytokine release and protection from premature ovarian failure in rodent models, so reduced *Ltf* may indicate impaired local antimicrobial/anti-inflammatory defense and altered granulosa–immune crosstalk [40]. *Sirpb1* receptors activate myeloid immune signaling via the DAP12–SYK–MAPK–NF-κB pathway, promoting inflammatory cytokine production [41]. Reduced *Sirpb1* expression suggests suppressed myeloid activation and inflammatory signaling in the ovary following DON exposure [41]. Similarly, our findings suggest that exposure to DON might attenuate the innate immune response and that causes ovarian cytotoxicity, which reduces the overall ovarian development and cleavage rate.

Interestingly, several of the affected hub genes are related to motile (9 + 2) cilia—the multiciliated, beating cilia that drive fluid/particle transport (classically in airway and fallopian tube/oviduct), and whose disruption causes primary ciliary dyskinesia (PCD)-type phenotypes [42,43]. Cilia are microtubule-based organelles that sense environmental cues and regulate key developmental and physiological processes; defects lead to ciliopathies [44]. *Rsph4a* is a radial spoke head component that helps transmit regulatory signals from the central pair to dynein motors to shape the ciliary beat. Loss disrupts motility/coordination [43]. *Drc1 (CCDC164)* is part of the nexin–dynein regulatory complex (N-DRC), a key scaffold that constrains microtubule sliding and regulates dynein activity for controlled beating [45]. *Zmynd10* is a dynein-arm assembly factor (cytoplasmic pre-assembly/chaperone relay); needed to build functional axonemal dynein arms, and its loss leads to cilia immotility/dynein arm defects [46]. *Hydin* is a central pair apparatus component (the “2” in 9 + 2) important for waveform control; defects can cause abnormal/stiff beating patterns [46]. Motile cilia in the infundibulum of the oviduct are essential for oocyte pickup (capturing the cumulus–oocyte complex after ovulation) and support gamete/embryo transport [47]. Interestingly, these hub genes related to motile cilia and axonemal structure (*Rsph4a*, *Drc1*, *Zmynd10*, *Hydin*, and *Tmem212*) were downregulated in both DON and DON + LEEK groups. Given the established roles of these genes in ciliary motility, their reduced expression raises the possibility that DON-induced stress may affect pathways related to ciliary structure or function. However, this interpretation remains speculative, as the present study did not directly assess ciliary structure or function. In the LKDON vs. DON comparison, motile cilia-related hub genes (*Dnah5*, *Dnah11*, *Tekt1*, *Zmynd10*, *Cfap44*, *Spag6l*, and *Nme9*) were predominantly upregulated, suggesting a partial restoration of cilia/axoneme-associated transcriptional programs [48]. This observation raises the possibility that co-treatment with fermented Chinese chive may modulate transcriptional pathways related to ciliary structure or function, although this hypothesis requires functional validation. In contrast, LKDON vs. CTRL showed reduced expression of *PTGS2* (COX-2) and several chemokine/cytokine mediators, indicating attenuation of DON-associated inflammation [49,50]. A key limitation of this study is the use of whole ovarian tissue, which precludes precise cellular localization of the observed transcriptional changes. Consequently, the functional implications of these cilia-related transcriptional changes remain to be determined. Future studies employing spatial or single-cell approaches, along with direct assessment of oviductal ciliary function and physiological mechanisms, are needed to validate the dose-dependent effects of fermented Chinese chive at escalating doses.

In the LEEK versus CTRL comparison, hub genes were uniformly upregulated and suggested an immune-priming ovarian response, highlighted by increased expression of *Il5*, *Cd27*, and *Crp*, genes associated with immune cell activation, survival, and innate inflammatory regulation [51,52,53]. This immune-skewed signature is biologically plausible in ovarian tissue because immune cells and cytokine networks contribute to ovarian homeostasis and remodeling across the reproductive cycle [54,55]. Overall, the LEEK-only hub-gene network supports an immunomodulatory rather than a stress-suppressive signature, aligning with previous reports that fermented Chinese chive (*Allium tuberosum*) exhibits antioxidant and immunomodulatory properties [56]. These findings suggest that fermented Chinese chive alone was associated with activation of immune regulatory pathways, whereas DON exposure is linked to reduced expression of immune activation genes. Moreover, Integrated physiological, transcriptomic, and chemical analyses indicate that fermented Chinese chive extract may mitigate DON-induced reproductive toxicity. UHPLC-QTOF-MS identified five flavonols, with kaempferol showing the highest abundance. Docking analysis suggested strong binding of kaempferol to PLA2G4B and RSPH4A, supporting its potential functional relevance. Although quercetin exhibited slightly stronger binding, the higher abundance of kaempferol highlights its likely contribution. These findings remain to be experimentally validated.

Our study showed Several limitations should need to explore, such as superovulation may have confounded outcomes by inducing inflammation and oxidative stress, and the absence of a natural-cycle control limits clear attribution of effects to DON. The cleavage rate reflects oocyte competence rather than overall fertility, and the 21-day exposure may not encompass a full folliculogenesis cycle. Reduced body weight without monitoring feed intake or using pair-fed controls introduces potential nutritional confounding. In addition, genetic variability in the Kunming mouse model may contribute to heterogeneous responses, and bulk transcriptomic analysis lacks cellular resolution. Future studies should incorporate natural-cycle conditions, dose–response and longer exposure designs, and controlled feeding strategies, alongside single-cell or spatial transcriptomic approaches, to better define cell-specific mechanisms and validate the impact of DON on ciliary motility and reproductive function.

## 5. Conclusions

This study demonstrates that deoxynivalenol impairs ovarian function by inducing oxidative imbalance, suppressing immune responses, and disrupting ciliary motility-related and other key pathways essential for oocyte quality. Fermented Chinese chive extract provides partial protection, primarily through modulation of immune and epithelial processes rather than complete restoration. These findings highlight a biologically relevant protective potential against deoxynivalenol and provide a basis for further investigation into its mechanisms and applications in reproductive health.

## Figures and Tables

**Table 1:** RT-qPCR primers used for validation.

Genes	Primer Sequences (5′-3′)	Product Length (bp)
*CCL11*	F: AGCTAGTCGGGAGAGCCTAC R: AAGGAAGTGACCGTGAGCAG	122
*ZMYND10*	F: GGGGCCTCCAGGTGGAATA R: GATGGAGGCCTCATGGTGTA	258
*PTGS2*	F: CATCCCCTTCCTGCGAAGTT R: CATGGGAGTTGGGCAGTCAT	178
*RSPH4A*	F: TTGCTGTCCTTCGCTCCAAT R: AGTGCAACTGGCTCTTGTGT	232
*PLA2G4B*	F: GTAGTCGAGTGGTTCCCAGG R: TAGGGAGGGTGGTTGGTTCC	123
*CD27*	F: ACAGCTGCTCAGTGTGATCC R: GCTTCTCTGTGCCATGAGGT	283
*GAPDH*	F: AGGCTTGAGATGGCTCTTGC R: TGCCGTGGGTGGAATCATAC	148

**Table 2:** Effects of treatments on mouse zygote cleavage rate.

Groups	Number of Zygotes with Cleavage	Total Number of Zygotes Observed	Mean Cleavage Rate ± SD (%)	*p*-Value			
				CTRL	LEEK	DON	LKDON
CTRL	58	102	56.89 ± 14.94 ^a^	—	0.522	<0.001	0.029
LEEK	54	106	51.18 ± 9.92 ^ab^	0.522	—	0.001	0.136
DON	26	99	27.08 ± 9.61 ^c^	<0.001	0.001	—	0.004
LKDON	44	101	45.20 ± 11.79 ^b^	0.029	0.136	0.004	—

Effects of DON exposure and fermented Chinese chive extract on mouse zygote cleavage rate. Values are presented as the number of cleaved zygotes, total number of zygotes observed and mean cleavage rate ± SD (%). Different lowercase superscript letters indicate statistically significant differences among groups (*p* < 0.05), whereas identical letters indicate no significant difference (*p* > 0.05). *p*-values represent pairwise comparisons between groups.

## Data Availability

The raw data has been uploaded to NCBI with Bioproject accession no: PRJNA1431777.

## Supplementary Materials

The following supporting information can be downloaded at https://www.mdpi.com/article/10.3390/antiox15040442/s1, Table S1: Statistical transcriptome summary; Table S2: FPKM normalized data; Table S3: Functional KEGG analysis of DEGs of DON vs CTRL comparion; Table S4: Functional KEGG analysis of DEGs of LEEK vs CTRL comparion; Table S5: Functional KEGG analysis of DEGs of LKDON vs CTRL comparison; Table S6: Metabolomics profiling of fermented Chinese Chive.
